# Meta-Analysis of Circulating Cell-Free DNA’s Role in the Prognosis of Pancreatic Cancer

**DOI:** 10.3390/cancers13143378

**Published:** 2021-07-06

**Authors:** Jelena Milin-Lazovic, Petar Madzarevic, Nina Rajovic, Vladimir Djordjevic, Nikola Milic, Sonja Pavlovic, Nevena Veljkovic, Natasa M. Milic, Dejan Radenkovic

**Affiliations:** 1 Institute for Medical Statistics and Informatics, Faculty of Medicine, University of Belgrade, 11000 Belgrade, Serbia; jelena.milin@med.bg.ac.rs (J.M.-L.); petar.madzarevic@gmail.com (P.M.); nina.rajovic@med.bg.ac.rs (N.R.); milic.natasa@mayo.edu (N.M.M.); 2 Department of Surgery, University Clinical Center of Serbia, 11000 Belgrade, Serbia; vladimir.djordjevic@kcs.ac.rs; 3 Faculty of Medicine, University of Belgrade, 11000 Belgrade, Serbia; nmilic1996@gmail.com; 4 Institute of Molecular Genetics and Genetic Engineering, University of Belgrade, 11000 Belgrade, Serbia; sonya@imgge.bg.ac.rs; 5 Vinca Institute of Nuclear Sciences, National Institute of the Republic of Serbia, University of Belgrade, 11000 Belgrade, Serbia; nevena.veljkovic@heliant.rs; 6 Heliant Ltd., 11000 Belgrade, Serbia; 7 Department of Internal Medicine, Division of Nephrology and Hypertension, Mayo Clinic, Rochester, MN 55902, USA

**Keywords:** cell-free DNA, pancreatic ductal adenocarcinoma, survival, meta-analysis

## Abstract

**Simple Summary:**

Pancreatic cancer is an aggressive disease with a poor prognosis. The analysis of cell-free DNA (cfDNA) for genetic abnormalities is a promising new approach for the diagnosis and prognosis of pancreatic cancer patients. In this study, we conducted a systematic review and meta-analysis of studies that reported cfDNA in pancreatic ductal adenocarcinoma (PDAC). In total, 48 studies were included in the qualitative synthesis, while 44 were assessed in the quantitative synthesis, including 3524 PDAC patients. An overall negative impact of cfDNA and *KRAS* mutations on the overall (OS) and progression free survival (PFS) (HR = 2.42, 95% CI: 1.95–2.99 and HR = 2.46, 95% CI: 2.01–3.00, respectively) were found. The performance of molecular studies to assess the presence of *KRAS* mutation by liquid biopsy may support global efforts to improve outcomes for PDAC patients.

**Abstract:**

Introduction: The analysis of cell-free DNA (cfDNA) for genetic abnormalities is a promising new approach for the diagnosis and prognosis of pancreatic cancer patients. Insights into the molecular characteristics of pancreatic cancer may provide valuable information, leading to its earlier detection and the development of targeted therapies. Material and Methods: We conducted a systematic review and a meta-analysis of studies that reported cfDNA in pancreatic ductal adenocarcinoma (PDAC). The studies were considered eligible if they included patients with PDAC, if they had blood tests for cfDNA/ctDNA, and if they analyzed the prognostic value of cfDNA/ctDNA for patients’ survival. The studies published before 22 October 2020 were identified through the PubMED, EMBASE, Web of Science and Cochrane Library databases. The assessed outcomes were the overall (OS) and progression-free survival (PFS), expressed as the log hazard ratio (HR) and standard error (SE). The summary of the HR effect size was estimated by pooling the individual trial results using the Review Manager, version 5.3, Cochrane Collaboration. The heterogeneity was assessed using the Cochran Q test and I^2^ statistic. Results: In total, 48 studies were included in the qualitative review, while 44 were assessed in the quantitative synthesis, with the total number of patients included being 3524. Overall negative impacts of cfDNA and *KRAS* mutations on OS and PFS in PDAC (HR = 2.42, 95% CI: 1.95–2.99 and HR = 2.46, 95% CI: 2.01–3.00, respectively) were found. The subgroup analysis of the locally advanced and metastatic disease presented similar results (HR = 2.51, 95% CI: 1.90–3.31). In the studies assessing the pre-treatment presence of *KRAS*, there was a moderate to high degree of heterogeneity (I^2^ = 87% and I^2^ = 48%, for OS and PFS, respectively), which was remarkably decreased in the analysis of the studies measuring post-treatment *KRAS* (I^2^ = 24% and I^2^ = 0%, for OS and PFS, respectively). The patients who were *KRAS* positive before but *KRAS* negative after treatment had a better prognosis than the persistently *KRAS*-positive patients (HR = 5.30, 95% CI: 1.02–27.63). Conclusion: The assessment of *KRAS* mutation by liquid biopsy can be considered as an additional tool for the estimation of the disease course and outcome in PDAC patients.

## 1. Introduction

Pancreatic cancer is an aggressive disease with a poor prognosis. Despite the constantly evolving therapeutic and diagnostic techniques, the survival rate for pancreatic cancer still remains low compared to other malignant tumors [1]. According to the American Cancer Society (ACS) and the National Cancer Institute (NCI), the overall 5-year survival rate for pancreatic cancer is below 9% [2]. Even the small percentage of people diagnosed with the local disease (10%) experience an aberrant 5-year survival rate of 37%. The vast majority see a fate of being diagnosed at the distant stage of the disease (53%), where the survival rate is 3% [2]. Pancreatic cancer’s low survival rates are attributed to late diagnosis, the lack of effective chemotherapy, and surgical limitations [3]. In 2017, there were 447,700 new cases diagnosed worldwide, and 441,082 deaths due to pancreatic cancer were recorded in the same year [4,5]. Pancreatic cancer accounts for 1.8% of all cancers, but causes 4.6% of all cancer deaths, thus resulting in it being the seventh highest cause of cancer death worldwide [2].

Cell-free DNA (cfDNA) has gained attention as a potential biomarker for a large variety of malignancies (lung, breast, liver, etc.) due to the increased levels of apoptosis, necrosis, pyroptosis, mitotic catastrophes, autophagy and phagocytosis present in cancer patients [6]. Thus, the detection of cfDNA changes in serum or plasma and the uncovering of genetic abnormalities being released from malignant tumors has been considered as promising candidate technique for cancer diagnosis through liquid biopsy [6,7]. The analysis of cell-free DNA (cfDNA) for genetic abnormalities is also a new promising approach for the diagnosis and prognosis of pancreatic cancer patients. Insights into the molecular characteristics of the pancreatic cancer may provide valuable information, leading to its earlier detection and the development of targeted therapies. The identification of a circulating biomarker for pancreatic cancer, in a non-invasive manner, is an exciting area of exploration, which may lead to personalized prognosis and therapeutic optimization from simple blood tests [8]. Previous studies have suggested that the vast majority of pancreatic ductal adenocarcinoma (PDAC) harbor mutations in the *KRAS* gene, with cfDNA mutant *KRAS* being an early marker of disease recurrence [9,10]. Tumor-derived cfDNA, known as circulating tumor DNA (ctDNA), has been the subject of extensive research. However, ctDNA’s clinical usability still has not been established due to the non-standardized technique for its quantification. With the introduction of digital droplet polymerase chain reaction (ddPCR), new insights in this area have been acquired [11,12]. ddPCR’s ability to aid in the determination of cfDNA and ctDNA’s size and level has been shown to yield prognostic value in pancreatic cancer [13]. In this study, we performed (1) a systematic review incorporating prior studies that explored the association between cfDNA and the prognosis of patients with PDAC, and (2) a meta-analysis which quantifies the association between the presence of *KRAS* mutation and overall survival (OS) and progression-free survival (PFS) in these patients.

## 2. Material and Methods

A systematic review was performed in accordance with the Preferred Reporting Items for Systematic Reviews [14] and the Meta-analysis of Observational Studies in Epidemiology [15]. The standardized protocol was specifically developed for the purpose of this review, and was used by independent reviewers.

### 2.1. Study Selection

The publications were screened for inclusion in the systematic review in two phases, and all of the disagreements were resolved by discussion at each stage with the inclusion of a third reviewer or by consensus. Studies were included based on the following criteria: (1) studies including patients with pancreatic cancer, (2) studies with blood tests for cfDNA/ctDNA, and (3) studies analyzing the prognostic value of cfDNA/ctDNA for patients’ survival results. Articles containing any of the following were excluded: (1) cfDNA/ctDNA extracted from tumor tissue; (2) studies without survival outcomes, such as OS and PFS; (3) studies lacking key data for the extraction of HR; or (4) diagnostic articles.

### 2.2. Search Strategy

A biostatistician with expertise in conducting systematic reviews and meta-analyses (N.M.M.) and a pancreatic cancer surgeon (D.R.) developed the search strategy. Searches of the PubMed, EMBASE, Web of Science and Cochrane Library databases until 22 October 2020 were performed for studies containing key words for cfDNA and pancreatic cancer: “cell-free DNA” or “ctDNA” or “cfDNA” or “circulating DNA” or “circulating tumor DNA” or “*KRAS*”, and “pancreatic cancer” or “pancreatic carcinoma” or “pancreatic adenocarcinoma”. There were no restrictions on the publication language or status. The authors of relevant studies were contacted in an attempt to obtain missing data, and to confirm the information on the study methodology and the results. The authors of relevant abstracts were contacted in order to identify eligible unpublished datasets. Reference lists of the articles that are included in the analysis were searched manually, as well as relevant reviews and editorials. Experts in the field were asked to provide information on potentially eligible studies.

### 2.3. Article Screening and Selection

Two reviewers (J.M.L., P.M.) independently evaluated the eligibility of all of the titles and abstracts, and performed full-text screening according to the inclusion and exclusion criteria. Disagreements were resolved by consensus (J.M.L., P.M.) or arbitration (N.M.M, D.R.).

### 2.4. Data Abstraction and Quality Assessment

Two reviewers (J.M.L., P.M.) independently extracted the following data: the first author’s name, year of publication, country, number of patients, study design, inclusion and exclusion criteria, TNM stage, sample origin, time of the sample collected, methods of DNA detection, detection markers, and information needed to assess the articles’ quality. The authors were contacted to clarify and confirm the accuracy of the abstracted data. The extraction of the survival outcome data included OS, PFS, disease-free survival (DFS), recurrence-free survival (RFS) and disease-specific survival (DSS). Hazard ratios (HR) with a corresponding 95% confidence interval (95% CI) were also obtained from the related articles.

### 2.5. Risk of Bias

The risk of bias in the individual studies was assessed according to the following criteria proposed by the GRADE (Grading of Recommendations, Assessment, Development and Evaluations) Working Group [16]: (1) failure to develop and apply appropriate eligibility criteria (the inclusion of a control population), (2) flawed measurements of both exposure and outcome, (3) failure to adequately control for confounding variables, and (4) incomplete follow-up. Two reviewers (J.M.L., P.M.) independently evaluated the risk of bias within and across the studies, and the overall quality of the gathered evidence. An adapted version of the Newcastle–Ottawa tool for observational studies was used [17].

### 2.6. Statistical Analysis

The assessed outcomes were OS and PFS expressed as the log HR and standard error (SE). For articles without explicit data for the HR and 95% CI, the logHR and SE were calculated by extracting the survival rates from Kaplan Meier curves using the WebPlotDigitizer v4.4 [18]. The HR was than estimated using a calculator formulated by Tierney et al. [19]. The number of patients at risk was extracted when available; if not, the numbers were calculated taking into account the total number of patients included in the survival analysis and selected time points accounting for the censored data [6,20,21,22,23,24,25,26,27,28,29,30,31]. In addition, if the HR data were not available, but were presented in the individual-level data, the HR with corresponding 95% CI were calculated by IBM SPSS, version 25 [32]. The summary HR effect size was estimated by pooling the individual trial results using the Review Manager, version 5.3, Cochrane Collaboration. The heterogeneity was assessed using the Cochran Q test and I^2^ statistic. According to Higgins and Thompson [33], the heterogeneity was defined as I^2^ > 50% or *p* value < 0.10. A random effect model was used due to presence of heterogeneity in all of the analysis [33]. The weight of each study was calculated by the inverse variance method and adjusted by effect models, which determined how much each study contributed to the pooled HR. Sensitivity analyses were performed in order to evaluate the effect of the sample origin and different survival outcome measures. A subgroup analysis was performed for locally advanced and metastatic disease. A separate forest plot was constructed for each analysis showing the HR (box), 95% CI (lines) and weight (size of box) for each trial. The diamond presented the overall effect size. The presence of publication bias was assessed by a linear regression test of the funnel plot asymmetry. *p* < 0.05 was considered to be statistically significant.

## 3. Results

### 3.1. Systematic Review

**Figure 1:**
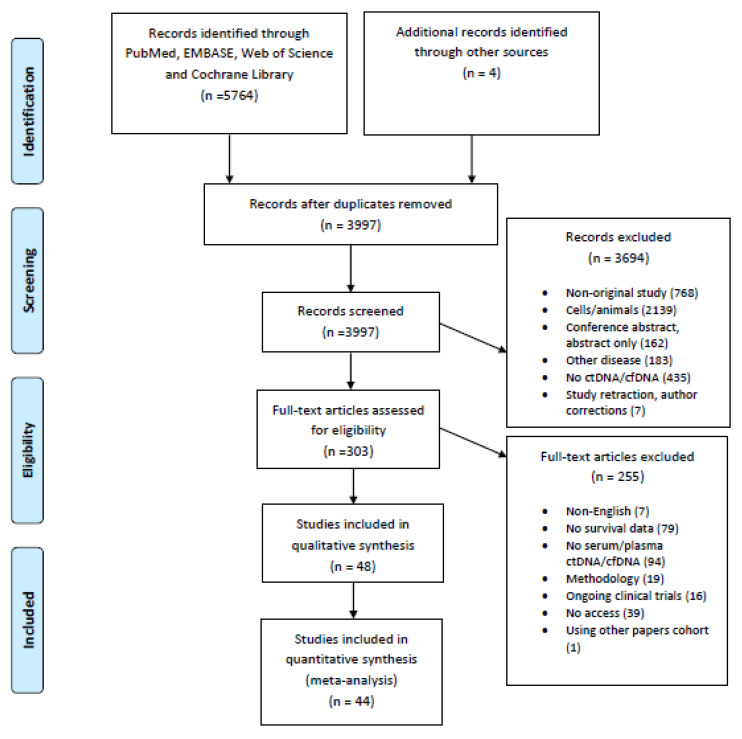
Flow chart of the study selection.

A total of 5768 potentially eligible articles were found. After duplicates were removed, 3997 titles and abstracts were screened. After reading the titles and abstracts, 3694 articles were excluded because they were not original studies, examined populations other than humans (animals, cell lines), examined other diseases, did not measure ctDNA/cfDNA, or were retracted studies, author corrections or abstracts. Of the 303 reviewed full text articles, 255 were excluded because they were not written in the English language, had no survival data, had no liquid biopsy data, were methodological studies, were ongoing clinical trials, or the full-text version of the article was not available. A total of 48 articles were selected for inclusion in the systematic review, and 44 studies were included in the meta-analysis. The flow chart presenting the steps of the study selection in detail is shown in Figure 1.

**Figure 2:**
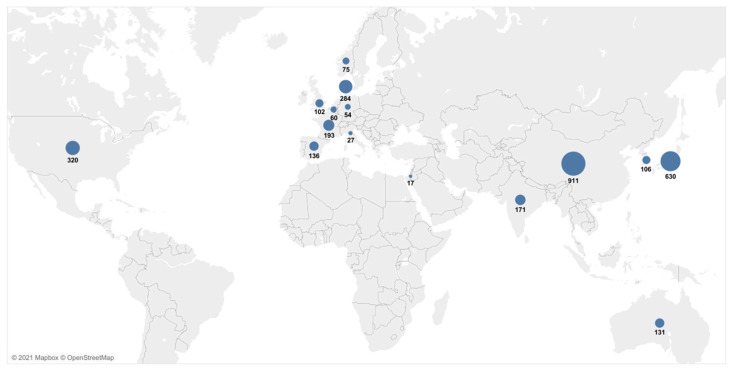
Geographical overview of the patient cases included in the meta-analyses. Data from multicenter and multicountry studies were excluded.

The characteristics of all of the 48 publications included in the systematic review are presented in detail in Table 1. Most of the studies were conducted in China or Japan (Figure 2). The studies were published between 1996 and 2020, with a minimum sample size of 10 [20] and a maximum of 210 patients [34]. The UICC/AJCC TNM classification was given by an exact number of patients in each stage in 18 of the studies [23,25,28,30,34,35,36,37,38,39,40,41,42,43,44,45,46,47,48], while the UICC/AJCC TNM classification was not reported for a subgroup of patients for which the ctDNA was measured, but was instead given for a total number of patients included in the study in four studies [20,49,50,51]. In total, 42 studies measured the cfDNA in plasma [9,13,21,24,25,26,27,28,29,30,31,34,35,36,38,39,40,41,42,43,44,45,46,48,49,50,51,52,53,54,55,56,57,58,59,60,61,62,63,64,65,66], three studies measured it from serum [37,47,67], two studies measured it from blood [20,22] and one study examined the cfDNA both in serum and plasma [23]. The time of the sampling was pre-treatment in 29 studies [9,13,23,24,26,29,34,36,37,38,39,40,43,44,46,49,52,53,54,55,57,58,60,62,63,64,65,66,67], pre/post-treatment in 10 studies [25,27,28,30,35,41,42,45,47,59], post-treatment only in two studies [48,50], pre/post and during treatment in one study [22], and six studies did not report the time of sampling [20,21,31,51,56,61]. *KRAS* was explicitly measured in 41 studies [9,20,21,22,23,24,25,26,27,28,29,30,31,34,35,36,37,38,39,41,42,43,44,45,46,47,48,49,50,52,53,54,55,57,58,59,60,61,62,63,64,67], the cfDNA/ctDNA total concentration was measured in four studies [13,52,56,65], cfDNATFx was measured in one study [66], hypermethylation was measured in one study [40], TP53 was measured in one study [62], ERBB exon 17 was measured in one study [58], and SPARC MI, UCHL1 MI, PENK M and NPTX2 MI were measured in one study [51].

A total of 44 studies used polymerase chain reaction (PCR) (ddPCR in 24 [9,23,24,25,26,28,30,31,34,37,38,41,44,45,46,49,52,55,57,58,59,60,63,67]; two used restriction fragment length-PCR (RFLP-PCR) [36,54]; two used nested PCR [53,56]; two used peptide nucleic acid-mediated clamping (PNA clamping) [22,47]; three used beads, emulsions, amplification and magnetics (BEAMing) [27,50,64]; one used mutant allele-specific PCR (MASA PCR) [35]; one used amplification-refractory mutation system PCR (ARMS PCR) [21]; one used quantitative methylation specific polymerase chain reaction (qMSP PCR) [51]; and an explicit PCR method was not reported in eight studies [20,29,39,40,42,46,48,66]). Next-generation sequencing (NGS) was used in three studies [61,62,65], and a bioassay was used as a primary method in one study [13].

### 3.2. Pre-Treatment KRAS Mutation, and Overall and Progression-Free Survival

**Figure 3:**
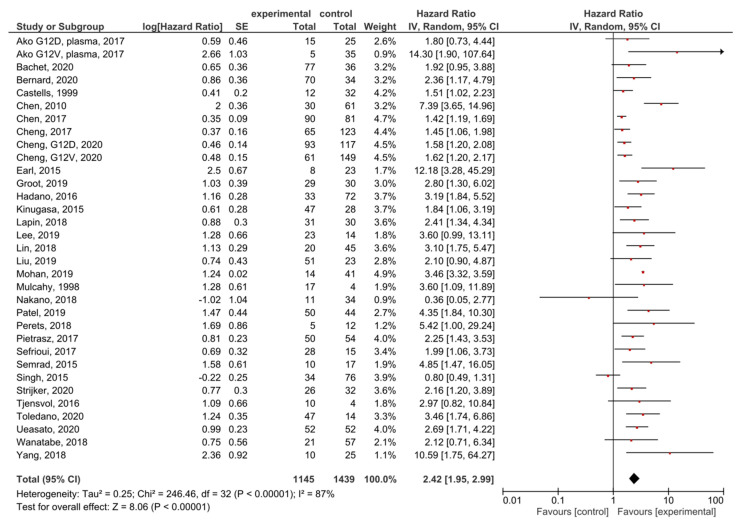
Forest plot presenting the relationship between the presence of *KRAS* mutations before treatment in PDAC patients and OS.

A meta-analysis was performed in order to assess the relationship between the presence of *KRAS* mutations in PDAC patients and OS before treatment. A total of 35 studies had OS as an outcome. Four studies were excluded from the overall HR effect size calculation due to measuring hypermethylation in ctDNA [40] or only post-treatment cfDNA, [48,50] or performing cfDNA TFx analysis [66]. Finally, 31 studies were included in the meta-analysis. The presence of pre-treatment *KRAS* mutations had significant prognostic value for OS in PDAC (HR = 2.42, 95% CI: 1.95–2.99) (Figure 3). There was a high degree of heterogeneity in the OS analysis (I^2^ = 87%) and a significant presence of publication bias (*p* = 0.021) (Supplemental Figure S1). The sensitivity analysis, excluding two studies which examined ctDNA in serum, showed a similar HR (HR = 2.49, 95% CI: 2.00–3.10) (Supplemental Figure S2).

**Figure 4:**
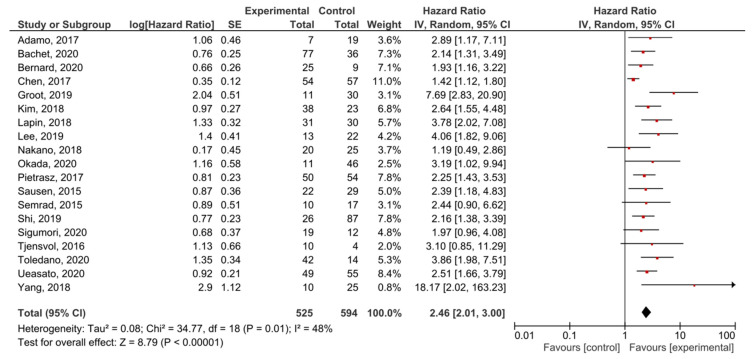
Forest plot presenting the relationship between the presence of *KRAS* mutations before treatment in PDAC patients and PFS.

A meta-analysis was performed in order to assess the relationship between the presence of *KRAS* mutations in PDAC patients and PFS before treatment. A total of 19 studies had PFS, DFS, RFS or DSS as an outcome. The presence of pre-treatment *KRAS* mutations demonstrated a significant prognostic value for PFS in PDAC patients (HR = 2.46, 95% CI: 2.01–3.00, *n* = 19) (Figure 4). There was a high degree of heterogeneity in the PFS analysis (I^2^ = 48%) %) and a significant presence of publication bias (*p* < 0.001) (Supplemental Figure S3). The sensitivity analysis including only PFS as an outcome resulted in a similar HR (HR = 2.27, 95% CI: 1.83–2.82, *n* = 14) (Supplemental Figure S4).

### 3.3. Post-Treatment KRAS Mutation and Overall and Progression-Free Survival

**Figure 5:**
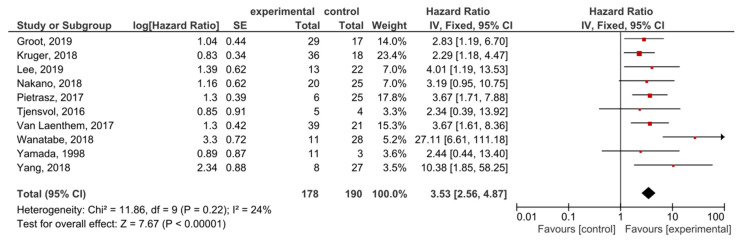
Forest plot presenting the relationship between the presence of *KRAS* mutations after treatment in PDAC patients and OS.

A total of 10 studies examined ctDNA post-treatment; the presence of post-treatment *KRAS* mutations demonstrated significant prognostic value for OS in PDAC patients (HR = 3.53, 95% CI: 2.56–4.87, *n* = 10) (Figure 5). There was a low degree of heterogeneity in the OS analysis (I^2^ = 24%) and no publication bias (*p* = 0.186) (Supplemental Figure S5). Patients in nine studies underwent different regimes of chemotherapy; in six studies, surgery was performed, and combined radiotherapy was performed in one study.

**Figure 6:**
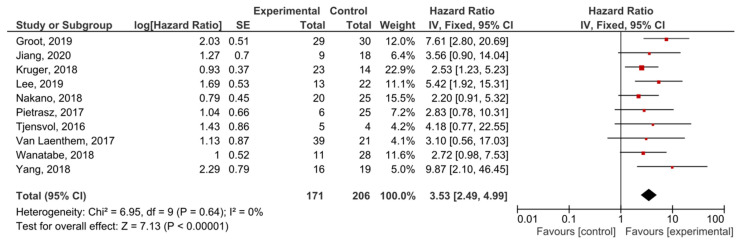
Forest plot presenting the relationship between the presence of *KRAS* mutations after treatment in PDAC patients and PFS.

The presence of post-treatment *KRAS* mutations demonstrated significant prognostic value for PFS in PDAC patients (HR = 3.53, 95% CI: 2.49–4.99, *n* = 10) (Figure 6). There was no heterogeneity in the PFS analysis (I^2^ = 0%) and no publication bias (*p* = 0.247) (Supplemental Figure S6). Patients in nine studies underwent different regimes of chemotherapy, and in six studies surgery was performed.

Changes in cfDNA positivity during the treatment with PFS as an outcome were examined in three studies. The responders (patients who were *KRAS* positive before treatment and *KRAS* negative after treatment) had a better prognosis than the non-responders (patients who were *KRAS* positive before treatment and remained *KRAS* positive after the treatment) (HR = 5.30, 95% CI: 1.02–27.63, *n* = 3) (Supplemental Figure S7).

### 3.4. Analysis of the Locally-Advanced and Metastatic Disease

**Figure 7:**
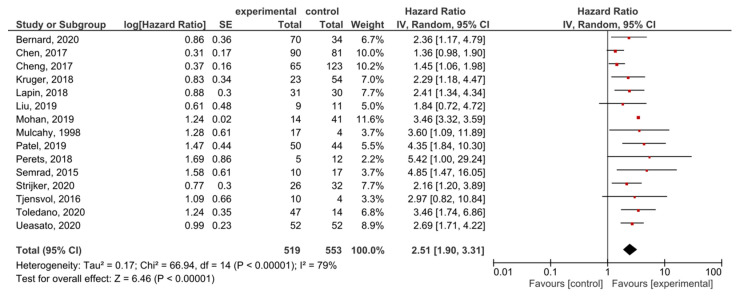
Forest plot presenting the relationship between the presence of *KRAS* mutations before treatment in locally-advanced and metastatic PDAC patients and OS.

A subgroup analysis of the studies examining the locally advanced and metastatic PDAC showed that *KRAS* mutations had significant prognostic value for OS (HR = 2.51, 95% CI: 1.90–3.31, *n* = 15) (Figure 7). There was a high degree of heterogeneity in the OS analysis (I^2^ = 79%) but no publication bias (*p* = 0.061) (Supplemental Figure S8). In the analysis examining only the metastatic disease, the effect was similar (HR = 1.90, 95% CI: 1.39–2.61, *n* = 6) (Supplemental Figure S9).

**Figure 8:**
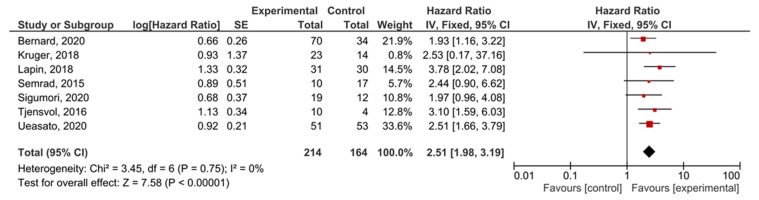
Forest plot presenting the relationship between the presence of *KRAS* mutations before treatment in locally-advanced and metastatic PDAC patients and PFS.

A subgroup analysis of the studies examining locally-advanced and metastatic PDAC showed that *KRAS* mutations demonstrated significant prognostic value for PFS (HR = 2.51, 95% CI: 1.98–3.19, *n* = 7) (Figure 8). There was not enough data to perform a separate analysis of metastatic disease with PFS as an outcome, or to test the funnel plot asymmetry (Supplemental Figure S10).

## 4. Discussion

In this study, we found an overall negative impact of *KRAS* mutations on OS and PFS in PDAC (HR = 2.42, 95% CI: 1.95–2.99 and HR = 2. 46, 95% CI: 2.01–3.00, respectively). The subgroup analysis of locally-advanced and metastatic disease presented similar results (HR = 2.51, 95% CI: 1.90–3.31). In studies assessing the pre-treatment presence of *KRAS* mutations, there was a high degree of heterogeneity in OS (I^2^ = 87%) and a moderate level of heterogeneity in the PFS analysis (I^2^ = 48%), which was remarkably decreased in the analysis of studies measuring post-treatment *KRAS* mutations (I^2^ = 24% and I^2^ = 0%, for OS and PFS, respectively).

There is a constant effort to find novel biomarkers which could improve the diagnosis, follow-up and therapeutic approaches in pancreatic cancer. The discovery that nucleic acids originating from cancer cells can be found in the peripheral circulation of cancer patients has had a major impact towards the development of non-invasive techniques, such as liquid-biopsy methodology, for the detection of tumor biomarkers. The analysis of cell-free DNA (cfDNA) for genetic abnormalities is a new promising research area for the diagnosis and prognosis of pancreatic cancer patients. CfDNA is also found in the blood of healthy individuals due to the continuous apoptosis/necrosis of hematopoietic cell line cells [6,68]. It usually consists of short fragments of less than 1000 base pairs (bp), with most being under 200bp [69]. When cell-free DNA originates from cancer cells, it is denoted as circulated tumor DNA (ctDNA). CtDNA is released into circulation primarily by the apoptosis of tumor cells and/or as a result of tumor necrosis [70,71]. Due to CtDNA’s extremely low concentration (as low as 0.01% of total cfDNA) and its fragmented and short-sized nature, the detection of the mutational status of ctDNA is very challenging, and highly sensitive techniques have to be utilized for its detection.

Different techniques are available for cfDNA/ctDNA detection: NGS, ddPCR, BEAMing, RFLP-PCR, and nested PCR, etc. For the mutational screening of cfDNA/ctDNA, the next-generation sequencing method (NGS) has been usually applied (both targeted and whole-genome sequencing). As the quantification of tumor-specific mutations in ctDNA has been shown to be more relevant for studying tumors, DdPCR was the most common technique used in the published studies due to its high sensitivity in the detection of rare mutations, its ability to quantify copy number variations and specific genomic loci, as well as its relatively simple workflow, in contrast to other methods [71]. Similar to the conventional PCR, this technology uses Taq-polymerase and primers/probes, but before the amplification reaction itself, the sample is divided into particles (”partitioning”)—tens of thousands of droplets—and the PCR reaction takes place in each of them. Another difference from conventional or real-time (qPCR) is that it is possible to perform the direct quantification of the PCR product, without using a standard curve. The primary applications for ddPCR are rare allele detection in heterogeneous samples like liquid biopsies or FFPE samples of solid tumors, non-invasive prenatal diagnostics, viral load detection, gene expression and copy number variation, single cell gene expression profiling, and the validation of low-frequency mutations identified by sequencing analysis. Moreover, epigenomic markers originating from tumor cells could be analyzed (methylation sites, circulating regulatory RNAs) [72]. A good agreement between BEAMing and ddPCR has been shown, with a kappa value of 0.91 (95% CI: 0.85–0.95) [73]. Recent advances in NGS technology have enabled similar sensitivity to the detection of ctDNA by ddPCR [74]. As each presented molecular platform has advantages and disadvantages, without evidence of a clear advantage for all of the purposes [28], the choice of platform should be determined to best meet the scientific and clinical questions being posed.

Most studies included measurements of ct/cf DNA in plasma, as plasma has been the preferred source for the extraction of circulating DNA. Even though serum contains a much higher amount (approximately a 2–24-times higher amount) of cfDNA than plasma, serum is not favored due to the possibility of contamination from white blood cells during clotting [75,76]. In this study, the sensitivity analysis excluding studies which used serum for cfDNA/ctDNA detection demonstrated similar results to those including only plasma measurements (HR = 2.49, 95% CI: 2.00–3.10).

Previous research has shown that the decrease in the levels of ctDNA during the treatment of PDAC patients may be a result of a significant reduction in the tumor burden. In contrast, the increase of the postoperative ctDNA levels may be due to a ctDNA release caused by tissue damage during surgery. Levy et al. showed that, in patients with PDAC, an endoscopic ultrasound fine-needle aspiration may be associated with increased an plasma concentration of cfDNA and the increased detection of mutant *KRAS* after the procedure [28]. Another reason for the increase in postoperative ctDNA levels may be a recurrence or tumor metastasis [47]. Lee et al. [42] suggested the importance of the post-operative analysis of ctDNA. Several of the studies included in this systematic review had pre/post treatment measurements of ctDNA, but only a few reported the survival between pre-positive/post-positive, pre-positive/post-negative, pre-negative/post-negative and pre-negative/post-positive patients. A meta-analysis of three studies that reported the survival between responders (pre-positive/post-negative) and non-responders (pre-positive/post-positive) presented poorer survival for persistently positive *KRAS* patients (HR = 5.30, 95% CI: 1.02–27.63, *n* = 3). Based on the main results of this meta-analysis, in terms of their survival prognosis, PDAC patients may be grouped in two categories: those who are ctDNA positive with worse outcomes, and those who are ctDNA negative with better outcomes [42,47,59]. In cases where the ctDNA is detectable at diagnosis but becomes undetectable post-treatment, a reduction in the relapse risk is present in comparison with those in whom the ctDNA remains detectable. CtDNA can provide valuable information to determine the treatment decisions stratifying patients at low and high risk of the progression and recurrence of the disease. Prospective research should be conducted based on standardized protocols in order to evaluate further treatment strategies. It was observed previously that, in the subset of patients with resectable PDAC, ctDNA may assist the clinician in the timely detection of recurrence and the concordant introduction/addition of therapeutic measures [42,77]. It should be noted that most of the studies from this systematic review included patients with varying disease stages, thus limiting the interpretation of the prognostic role of ctDNA in resectable disease, or as a marker of disease recurrence. The data collected was utilized to determine the ways in which ctDNA’s presence impacts the prognosis, rather than how specific ctDNA subtypes impact the prognosis or at what stage in the disease/treatment course these prognostic predictors are valid. Wild-type alternative and other onco-drivers present in cfDNA in specific patient cohorts (ex. *KRAS* G12C) are known to be highly actionable, allowing for precision medicine [78].

RAS genes (HRAS, *KRAS*, and NRAS) comprise the most frequently mutated oncogene family in human cancer. *KRAS* is mutated in 25% of all of the cancer cases, and is associated with poor disease prognosis [77]. Given that *KRAS* mutations are found in nearly all of the PDAC, this cancer type is arguably the most RAS-addicted cancer. Its roles in pancreatic cancer cell processes, such as increased proliferation, survival, migration and invasion, are well known [78]. An activating point mutation of the *KRAS* oncogene on codon 12 (exon 2) is the initiating event in the majority of PDAC cases (70–95%). *KRAS*^G12D^ and *KRAS*^G12V^ mutations constitute about 80% of the *KRAS* mutations in PDAC [79]. For decades, *KRAS* oncoprotein was classified as undruggable cancer target [77]. According to growing evidence linking *KRAS* mutations to increased PDAC growth, the National Cancer Institute identified the targeting of *KRAS* as one of four major priorities for pancreatic cancer research. Targeted therapies and *KRAS* inhibitors appear to be very promising. A recent review investigating small-molecule *KRAS* inhibitors suggested that combining the antitumor effects from innovative new *KRAS* inhibitors like AMG510 with other agents, nanoparticles, or other auxiliary processes that can overcome the PDAC biochemical and tissue delivery issues offers hope for a new therapeutic way forward in PDAC [80].

Recently, the significance of a multigene approach based on liquid biopsy was highlighted to guide individual tailored therapy for PDAC patients. Alterations in other driver genes such as *CDKN2A*, *BRCA1/2*, *ERB2* and *NTRK*, etc. have been shown to be associated with PDAC, and they are also relevant to targeted treatments. In the recent study by Pishvaian et al. presenting 1856 patients with PDAC, 58% of the patients had molecular testing, actionable molecular alterations were identified in 26%, out of which 46 patients received a matched therapy as a second- or later-line therapy and presented a better OS [81]. In a study including 259 PDAC patients with varying disease stages, a potentially actionable mutation was detected in 29% [82], while in a study including patients with advanced PDAC, therapeutically relevant alterations were observed in 48% of the samples [83]. Given the difficulties that exist in obtaining a tumor sample in PDAC, the results of these studies highlight the importance of performing molecular profiling based on liquid biopsy, due to its simplicity and accessibility, and the importance of finding actionable early mutations in a tumor with limited therapeutic options. In addition, given that mutations may vary during the course of the disease, it is important to monitor these molecular changes [84]. With the ongoing debate regarding the use of neoadjuvant therapy in purely resectable PDAC patients, it should be also noted that ctDNA detection may play a relevant role in answering a key question: who, from these particular groups of patients, is a candidate for neoadjuvant therapy?

A strength of this study was the broad sensitive search strategy used across multiple bibliographic databases that resulted in 3997 articles screened and 48 studies included in the systematic review. The most recent meta-analysis of similar scope started with an initial set of 724 articles, with the inclusion of 18 articles due to its narrow specific search strategy [10]. The greatest number of patients (*n* = 3524) included in this analysis generated the most comprehensive meta-analysis of the assessment of the prognostic utility of cfDNA/ctDNA’s in PDAC, while the meta-analysis assessing *KRAS* mutations included a total 2400 patients. In addition, in order to increase the utility of the data with were not directly shown, but were available in figures or as individual data, we used several recommended techniques to obtain HRs.

This study had several limitations, related to the clarification of liquid biopsy results in general, and those related to the proper understanding of the meta-analyses’ results. The accurate interpretation of liquid biopsy results is rather challenging because of the presence of somatic mosaicism in plasma. One of the most common sources of the biological background noise of blood liquid biopsy is somatic mutation in blood cells [85]. The accumulation of somatic mutations in hematopoietic stem cells leads to their clonal expansion. This process, called clonal hematopoiesis (CH) is common in an aging healthy population [86]. Interestingly, not only mutations related to hematological malignancies, but also mutations in genes characteristic for solid tumors are detected as a result of CH. Mutations in the *KRAS* gene are also found as CH-mutations [87]. It is very important to exclude these non-tumor derived CH-mutations, in order to avoid the incorrect interpretation and inappropriate therapeutic management of solid tumors. CH mutations can be determined by performing the paired sequencing of plasma cfDNA and DNA from white blood cells. It is expected that artificial intelligence tools, such as machine learning, will enable the distinction between CH mutations and tumor-derived molecular alterations in liquid biopsy [85].

A relatively large number of studies were included, resulting in a wide range of initial tumor burdens, mixed-size patient groups and various methods of ctDNA detection, all of which contributed to increased heterogeneity. Different therapies, study designs and a range of follow up times also contributed to this high value of heterogeneity. Specific conclusions based on tumor stage, ctDNA concentration and mutations other than *KRAS* were not possible to derive. The studies included in our meta-analyses encompassed, predominantly, patients from European and Asian populations (Figure 2). Given that the misclassification of the variants coming from data that did not include dissimilar subpopulations could potentially lead to the inadequate treatments of individuals from underrepresented populations [88], the conclusions derived here should be treated cautiously. Large-scale population studies indicated that there are more significant numbers of population-specific variations than we believed previously [89,90]. Thus, the potential of ctDNA to improve the health outcomes for PDAC patients should be evaluated in the context of various populations. The results of the meta-analysis presenting the relationship between the presence of *KRAS* mutations before treatment and the survival of PDAC patients should be interpreted with caution due to the presence of significant publication bias.

## 5. Conclusions

The assessment of *KRAS* mutation by liquid biopsy can be considered as an additional tool for the estimation of the disease course and outcome in PDAC patients. While ddPCR was utilized in most studies to detect the *KRAS* mutations, due to greater test sensitivity, other technologies in the era of NGS may also be useful in clinical practice. The choice of the molecular platform should be determined in order to best meet the scientific and clinical questions being posed.

## Figures and Tables

**Table 1:** Overview of the current literature on circulating tumor DNA in pancreatic cancer, with survival endpoints and patient group data.

Reference (Year)	Country	*n*	TNM or Other Tumor Stage Classification Available	Median Follow up *	Sample Origin	Time of Sample Origin	Method	Marker	Endpoints
Nomoto [20] (1996)	Japan	10	II-IV, numbers not reported	not reported	blood	not reported	PCR	KRAS	OS
Mulcahy [54] (1998)	England	21	21 unresectable disease	5.5 (1.2–16.7)	plasma	pre-treatment	RFLP-PCR	KRAS	OS
Yamada [35] (1998)	Japan	30	I = 3, II = 6, III = 7, IV = 5	12.2 (1.3–43.5)	plasma	pre/post treatment	MASA-PCR	KRAS codon 12	OS
Castells [36] (1999)	Spain	44	I = 4, II = 11, III = 5, IV = 23, unknown = 1	9 (6–17)	plasma	pre-treatment	RFLP-PCR	KRAS	OS
Chen [53] (2010)	China	91	unresectable	7 (3–21)	plasma	pre-treatment	Nested PCR	KRAS	OS
Earl [55] (2015)	Spain	31	resectable = 10, locally advanced = 8, metastatic = 13	not reported	plasma	pre-treatment	ddPCR	KRAS	OS
Kinugasa [37] (2015)	Japan	75	II = 2, III = 5, IV = 68	not reported	serum	pre-treatment	ddPCR	KRAS, G12V	OS
Sausen [49] (2015)	USA and Denmark	51	I–III, numbers not reported	32	plasma	pre-treatment	ddPCR	KRAS	PFS
Semrad [21] (2015)	USA	28	locally advanced and metastatic, numbers not reported	not reported	plasma	not reported	ARMS PCR	KRAS	OS, PFS
Singh [56] (2015)	India	110	resectable and unresectable, numbers not reported	not reported	plasma	not reported	Nested PCR	ctDNA concentration	OS
Hadano [38] (2016)	Japan	105	I–II = 84, III–IV = 21	54 (14–96)	plasma	pre-treatment	ddPCR	KRAS	OS
Tjensvoll [22] (2016)	Norway	14	locally advanced = 2, metastatic = 12	3.7	blood	pre/post and during treatment	PNA directed PCR clamping	KRAS B1, B2	OS, PFS
Adamo [57] (2017)	United Kingdom	26	resectable = 6, non-resectable = 5, metastatic = 15	not reported	plasma	pre-treatment	tNGS and ddPCR	KRAS	DSS
Ako [23] (2017)	Japan	40	UICC1 = 22, UICC2 = 7, UICC3 = 9, not clear = 2	7.8 (0.3–30.2)	plasma/serum	pre-treatment	ddPCR	KRAS, G12D, G12V	OS
Allenson [24] (2017)	USA, Czech and Slovakia	85	Localized = 33, localized postsurgical = 20, locally advanced = 13, metastatic = 19	not reported	plasma	pre-treatment	ddPCR	KRAS	OS
Chen [39] (2017)	Denmark	189	III = 40, IV = 149	not reported	plasma	pre-treatment	NGS and PCR	KRAS	OS
Cheng [58] (2017)	China	188	Metastatic = 188	no reported	plasma	pre-treatment	NGS/ddPCR	KRAS G12V, ERBB2, exon 17	OS
Del Re [25] (2017)	Italy	27	III = 4, IV = 23	not reported	plasma	pre/post treatment	ddPCR	KRAS	PFS
Henriksen [40] (2017)	Denmark	95	I = 11, II = 29, III = 13, IV = 42	not reported	plasma	pre-treatment	PCR	Hypermethylation	OS
Pietrasz [59] (2017)	France	135	resectable, locally advanced and stage IV	34.2	plasma	pre/post treatment	ddPCR/NGS	KRAS	OS, PFS
Sefrioui [26] (2017)	France	58	resectable = 16, locally advanced = 18, metastatic = 24	7.5 (1–64)	plasma	pre-treatment	ddPCR	KRAS ctDNA	OS
Van Laethem [50] (2017)	Belgium	60	II-IV, numbers not reported	not reported	plasma	post treatment	BEAMing	KRAS	OS
Kim [60] (2018)	Korea	106	resectable = 41, locally advanced = 25, metastatic = 40	10.3 (0.07–19.96)	plasma	pre-treatment	ddPCR	KRAS concentration	OS, PFS
Kruger [27] (2018)	Germany	54	locally advanced = 7, metastatic = 47	not reported	plasma	pre/post treatment	BEAMing	KRAS	OS, PFS
Lapin [13] (2018)	Norway	61	locally advanced = 6, metastatic = 55	7.7 months (0.3–25.8)	plasma	pre-treatment and during	Agilent 2100 Bioanalyzer and Agilent High Sensitivity DNA kit	cfDNA concentration and size	PFS, OS
Levy [28] (2018)	USA	35	I = 3, II = 16, III = 7, IV = 9	11.2 (5.48–13.2)	plasma	pre/post treatment	ddPCR	KRAS	OS
Lin [46] (2018)	China	65	UICCI/II = 5, UICCIII/IV = 60	21	plasma	pre-treatment	ddPCR	KRAS codon 12	OS
Nakano [47] (2018)	Japan	45	I = 2, II = 43	not reported	serum	pre/post treatment	PNA directed PCR clamping	KRAS codon12/13	OS, DFS
Perets [29] (2018)	Israel	17	metastatic = 14	not reported	plasma	pre-treatment	PCR	KRAS	OS
Yang [41] (2018)	China	35	I = 3, II = 29, III = 3	12.4 (6.1–17.2)	plasma	pre/post treatment	ddPCR	KRAS	OS, PFS
Bernard [52] (2019)	USA	104	Metastatic = 104	187 days	plasma	pre-treatment	ddPCR	ctDNA, KRAS	PFS
Groot [30] (2019)	USA	59	I–II = 43, III–IV = 16	16 (13–19)	plasma	pre/post treatment	ddPCR	KRAS	OS, PFS, RFR
Lee [42] (2019)	Australia, New Zealand and Singapore	131	I = 1, II = 3, III = 33	38.4	plasma	pre/post treatment	PCR	KRAS	RFS, OS
Liu [43] (2019)	China	112	I/II = 58, III/IV = 22	not reported	plasma	pre-treatment	PCR	KRAS	OS
Mohan [9] (2019)	England	55	locally advanced disease = 24, metastatic = 31	no reported	plasma	pre-treatment	NGS/ddPCR	KRAS	OS
Patel [61] (2019)	USA	94	Advanced = 94	18.2 (95% CI, 13.7–22.7).	plasma	not reported	NGS	KRAS	OS
Shi [44] (2019)	China	113	I = 49, II = 57, III = 7	23.6	plasma	pre-treatment	NGS/ddPCR	KRAS	RFS, OS
Watanabe [45] (2019)	Japan	78	locally advanced = 13, metastasis = 20, recurrence = 4, peritoneal dissemination = 2. I = 3, II = 33, III = 1, IV = 1	16.2	plasma	pre/post treatment	ddPCR	KRAS (C12V, G12D, G12R, and Q61H)	OS
Bachet [62] (2020)	Multicenter	113	advanced = 113	not reported	plasma	pre-treatment	NGS	KRAS, TP53	OS, PFS
Cheng [34] (2020)	China	210	III = 71, IV = 139	not reported	plasma	pre-treatment	ddPCR	KRAS G12V, G12D	OS
Jiang [48] (2020)	China	27	I = 13, II = 9, IV = 5	18.6 months (12.4–28.9)	plasma	post treatment	PCR/NGS	KRAS	DFS
Okada [31] (2020)	Japan	96	resectable = 66 unresectable = 30	not reported	plasma	not reported	ddPCR	KRAS	DFS
Singh [51] (2020)	India	61	I-IV, numbers not reported	36	plasma	not reported	qMSP PCR	SPARC MI, UCHL1 MI, PENK M, NPTX2 MI	OS
Strijker [63] (2020)	The Netherlands and Italy	58	no metastases on baseline imaging or distant lymph only = 10, liver metastases = 37, metastases other than liver = 10	12.3 (2.3–27.7)	plasma	pre-treatment	NGS/ddPCR	KRAS codon 12/13	OS
Sugimori [67] (2020)	Japan	47	locally advanced = 17, peritoneal metastasis = 9, liver/lung metastasis = 21	not reported	serum	pre-treatment	ddPCR/NGS	KRAS G12/13	PFS
Toledano [64] (2020)	Spain	61	distant metastasis = 61	not reported	plasma	pre-treatment	BEAMing	KRAS	OS, PFS
Uesato [65] (2020)	Japan	104	advanced and liver metastasis = 104	not reported	plasma	pre-treatment	NGS	ctDNA	OS, PFS
Wei [66] (2020)	China	70	no liver metastasis = 12, liver metastasis = 51	not reported	plasma	pre-treatment	WGS/PCR	ctDNATFx	OS

BEAMing, beads, emulsions, amplification and magnetics; ddPCR, digital droplet polymerase chain reaction; NGS, next-generation sequencing; PCR, polymerase chain reaction; RFLP-PCR, restriction fragment length polymorphism polymerase chain reaction; MASA-PCR, mutant allele-specific polymerase chain reaction; PNA-clamping PCR, peptide nucleic acid-mediated clamping polymerase chain reaction; NGS, next-generation sequencing; WGS, whole-genome sequencing; ARMS PCR, amplification-refractory mutation system; TFx, tumor fraction; OS, overall survival; RFR, relapse free rate; PFS, progress-free survival; DSS, disease specific survival; DFS, disease free survival; qMSP PCR, quantitative methylation specific polymerase chain reaction. * median follow up in months.

## Data Availability

All of the data generated in this research are in the manuscript or its supplemental files.

## Supplementary Materials

The following are available online at https://www.mdpi.com/article/10.3390/cancers13143378/s1, Figure S1: Funnel plot of the meta-analysis presented in Figure 3, Figure S2: Forest plot presenting the relationship between presence of KRAS mutations before treatment in PDAC patients and OS: sensitivity analysis excluding samples from serum, Figure S3: Funnel plot of the meta-analysis presented in Figure 4, Figure S4: Forest plot presenting the relationship between presence of KRAS mutations before treatment in PDAC patients and PFS: sensitivity analysis including only PFS but not DFS and DSS, Figure S5: Funnel plot of the meta-analysis presented in Figure 5, Figure S6: Funnel plot of the meta-analysis presented in Figure 6, Figure S7: Forest plot comparing PFS between responders (patients who were KRAS positive before treatment and KRAS negative after treatment) and non-responders (patients who were KRAS positive before treatment and remained KRAS positive after the treatment), Figure S8: Funnel plot of the meta-analysis presented in Figure 7, Figure S9: Forest plot presenting the relationship between presence of KRAS mutations before treatment in metastatic PDAC patients and OS, Figure S10: Funnel plot of the meta-analysis presented in Figure 8.

## References

[1] Van Cutsem E., Hidalgo M., Canon J.L., Macarulla T., Bazin I., Poddubskaya E., Manojlovic N., Radenkovic D., Verslype C., Raymond E. (2018). Phase I/II trial of pimasertib plus gemcitabine in patients with metastatic pancreatic cancer. Int. J. Cancer.

[2] American Cancer Society (2020). Cancer Facts & Figures 2020.

[3] Paulson A.S., Tran Cao H.S., Tempero M.A., Lowy A.M. (2013). Therapeutic Advances in Pancreatic Cancer. Gastroenterology.

[4] GBD 2017 Disease and Injury Incidence and Prevalence Collaborators (2018). Global, regional, and national incidence, prevalence, and years lived with disability for 354 diseases and injuries for 195 countries and territories, 1990–2017: A systematic analysis for the Global Burden of Disease Study 2017. Lancet.

[5] Institute for Health Metrics and Evaluation. http://ghdx.healthdata.org/gbd-results-tool.

[6] Bronkhorst A.J., Ungerer V., Holdenrieder S. (2019). The emerging role of cell-free DNA as a molecular marker for cancer management. Biomol. Detect Quantif..

[7] Kato S., Janku F. (2015). Cell-free DNA as a novel marker in cancer therapy. Biomark Med..

[8] Gall T.M.H., Belete S., Khanderia E., Frampton A.E., Jiao L.R. (2019). Circulating Tumor Cells and Cell-Free DNA in Pancreatic Ductal Adenocarcinoma. Am. J. Pathol..

[9] Mohan S., Ayub M., Rothwell D.G., Gulati S., Kilerci B., Hollebecque A., Sun Leong H., Smith N.K., Sahoo S., Descamps T. (2019). Analysis of circulating cell-free DNA identifies KRAS copy number gain and mutation as a novel prognostic marker in Pancreatic cancer. Sci. Rep..

[10] Chen L., Zhang Y., Cheng Y., Zhang D., Zhu S., Ma X. (2018). Prognostic value of circulating cell-free DNA in patients with pancreatic cancer: A systemic review and meta-analysis. Gene.

[11] Nygaard A.D., Holdgaard P.C., Spindler K.L., Pallisgaard N., Jakobsen A. (2014). The correlation between cell-free DNA and tumour burden was estimated by PET/CT in patients with advanced NSCLC. Br. J. Cancer.

[12] Hindson B.J., Ness K.D., Masquelier D.A., Belgrader P., Heredia N.J., Makarewicz A.J., Bright I.J., Lucero M.Y., Hiddessen A.L., Legler T.C. (2011). High-throughput droplet digital PCR system for absolute quantitation of DNA copy number. Anal. Chem..

[13] Lapin M., Oltedal S., Tjensvoll K., Buhl T., Smaaland R., Garresori H., Javle M., Glenjen N.I., Abelseth B.K., Gilje B. (2018). Fragment size and level of cell-free DNA provide prognostic information in patients with advanced pancreatic cancer. J. Transl. Med..

[14] Liberati A., Altman D.G., Tetzlaff J., Mulrow C., Gøtzsche P.C., Ioannidis J.P., Clarke M., Devereaux P.J., Kleijnen J., Moher D. (2009). The PRISMA statement for reporting systematic reviews and meta-analyses of studies that evaluate healthcare interventions: Explanation and elaboration. BMJ.

[15] Stroup D.F., Berlin J.A., Morton S.C., Olkin I., Williamson G.D., Rennie D., Moher D., Becker B.J., Sipe T.A., Thacker S.B. (2000). Meta-analysis of observational studies in epidemiology: A proposal for reporting. Meta-analysis of Observational Studies in Epidemiology (MOOSE) group. JAMA.

[16] Higgins J.P.T., Thomas J., Chandler J., Cumpston M., Li T., Page M.J., Welch V.A. (2019). Cochrane Handbook for Systematic Reviews of Interventions.

[17] Wells G.A., Shea B., O’Connell D., Peterson J., Welch V., Losos M., Tugwell P. (2014). The Newcastle–Ottawa Scale (NOS) for Assessing the Quality of Nonrandomisedstudies in Meta-Analysis.

[18] Cramond F., O’Mara-Eves A., Doran-Constant L., Rice A.S., Macleod M., Thomas J. (2018). The development and evaluation of an online application to assist in the extraction of data from graphs for use in systematic reviews. Wellcome Open Res..

[19] Tierney J.F., Stewart L.A., Ghersi D., Burdett S., Sydes M.R. (2007). Practical methods for incorporating summary time-to-event data into meta-analysis. Trials.

[20] Nomoto S., Nakao A., Kasai Y., Harada A., Nonami T., Takagi H. (1996). Detection of ras gene mutations in perioperative peripheral blood with pancreatic adenocarcinoma. Jpn. J. Cancer Res..

[21] Semrad T., Barzi A., Lenz H.J., Hutchins I.M., Kim E.J., Gong I.Y., Tanaka M., Beckett L., Holland W., Burich R.A. (2015). Pharmacodynamic separation of gemcitabine and erlotinib in locally advanced or metastatic pancreatic cancer: Therapeutic and biomarker results. Int. J. Clin. Oncol..

[22] Tjensvoll K., Lapin M., Buhl T., Oltedal S., Steen-Ottosen Berry K., Gilje B., Søreide J.A., Javle M., Nordgård O., Smaaland R. (2016). Clinical relevance of circulating KRAS mutated DNA in plasma from patients with advanced pancreatic cancer. Mol. Oncol..

[23] Ako S., Nouso K., Kinugasa H., Dohi C., Matushita H., Mizukawa S., Muro S., Akimoto Y., Uchida D., Tomoda T. (2017). Utility of serum DNA as a marker for KRAS mutations in pancreatic cancer tissue. Pancreatology.

[24] Allenson K., Castillo J., San Lucas F.A., Scelo G., Kim D.U., Bernard V., Davis G., Kumar T., Katz M., Overman M.J. (2017). High prevalence of mutant KRAS in circulating exosome-derived DNA from early-stage pancreatic cancer patients. Ann. Oncol..

[25] Del Re M., Vivaldi C., Rofi E., Vasile E., Miccoli M., Caparello C., d’Arienzo P.D., Fornaro L., Falcone A., Danesi R. (2017). Early changes in plasma DNA levels of mutant KRAS as a sensitive marker of response to chemotherapy in pancreatic cancer. Sci. Rep..

[26] Sefrioui D., Blanchard F., Toure E., Basile P., Beaussire L., Dolfus C., Perdrix A., Paresy M., Antonietti M., Iwanicki-Caron I. (2017). Diagnostic value of CA19.9, circulating tumour DNA and circulating tumour cells in patients with solid pancreatic tumours. Br. J. Cancer.

[27] Kruger S., Heinemann V., Ross C., Diehl F., Nagel D., Ormanns S., Liebmann S., Prinz-Bravin I., Westphalen C.B., Haas M. (2018). Repeated mutKRASctDNA measurements represent a novel and promising tool for early response prediction and therapy monitoring in advanced pancreatic cancer. Ann. Oncol..

[28] Levy M.J., Kipp B.R., Milosevic D., Schneider A.R., Voss J.S., Avula R., Kerr S.E., Henry M.R., Highsmith E., Liu M.C. (2018). Analysis of Cell-Free DNA to Assess Risk of Tumoremia Following Endoscopic Ultrasound Fine-Needle Aspiration of Pancreatic Adenocarcinomas. Clin. Gastroenterol. Hepatol..

[29] Perets R., Greenberg O., Shentzer T., Semenisty V., Epelbaum R., Bick T., Sarji S., Ben-Izhak O., Sabo E., Hershkovitz D. (2018). Mutant KRAS Circulating Tumor DNA Is an Accurate Tool for Pancreatic Cancer Monitoring. Oncologist.

[30] Groot V.P., Mosier S., Javed A.A., Teinor J.A., Gemenetzis G., Ding D., Haley L.M., Yu J., Burkhart R.A., Hasanain A. (2019). Circulating Tumor DNA as a Clinical Test in Resected Pancreatic Cancer. Clin. Cancer Res..

[31] Okada T., Mizukami Y., Ono Y., Sato H., Hayashi A., Kawabata H., Koizumi K., Masuda S., Teshima S., Takahashi K. (2020). Digital PCR-based plasma cell-free DNA mutation analysis for early-stage pancreatic tumor diagnosis and surveillance. J. Gastroenterol..

[32] IBM Corp (2017). Released 2017. IBM SPSS Statistics for Windows, Version 25.0.

[33] Higgins J.P.T., Thompson S.G. (2002). Quantifying heterogeneity in a meta-analysis. Stat Med..

[34] Cheng H., Luo G., Jin K., Fan Z., Huang Q., Gong Y., Xu J., Yu X., Liu C. (2020). Kras mutation correlating with circulating regulatory T cells predicts the prognosis of advanced pancreatic cancer patients. Cancer Med..

[35] Yamada T., Nakamori S., Ohzato H., Oshima S., Aoki T., Higaki N., Sugimoto K., Akagi K., Fujiwara Y., Nishisho I. (1998). Detection of K-ras gene mutations in plasma DNA of patients with pancreatic adenocarcinoma: Correlation with clinicopathological features. Clin. Cancer Res..

[36] Castells A., Puig P., Móra J., Boadas J., Boix L., Urgell E., Solé M., Capellà G., Lluís F., Fernández-Cruz L. (1999). K-ras mutations in DNA extracted from the plasma of patients with pancreatic carcinoma: Diagnostic utility and prognostic significance. J. Clin. Oncol..

[37] Kinugasa H., Nouso K., Miyahara K., Morimoto Y., Dohi C., Tsutsumi K., Kato H., Matsubara T., Okada H., Yamamoto K. (2015). Detection of K-ras gene mutation by liquid biopsy in patients with pancreatic cancer. Cancer.

[38] Hadano N., Murakami Y., Uemura K., Hashimoto Y., Kondo N., Nakagawa N., Sueda T., Hiyama E. (2016). Prognostic value of circulating tumour DNA in patients undergoing curative resection for pancreatic cancer. Br. J. Cancer.

[39] Chen I., Raymond V.M., Geis J.A., Collisson E.A., Jensen B.V., Hermann K.L., Erlander M.G., Tempero M., Johansen J.S. (2017). Ultrasensitive plasma ctDNA KRAS assay for detection, prognosis, and assessment of therapeutic response in patients with unresectable pancreatic ductal adenocarcinoma. Oncotarget.

[40] Henriksen S.D., Madsen P.H., Larsen A.C., Johansen M.B., Pedersen I.S., Krarup H., Thorlacius-Ussing O. (2017). Cell-free DNA promoter hypermethylation in plasma as a predictive marker for survival of patients with pancreatic adenocarcinoma. Oncotarget.

[41] Yang X., Xu W., Tian X., Wu J., Lv A., Li C., Guan X., Qian H., Hao C. (2018). Diagnostic and prognostic value of KRAS mutations in circulating pancreatic ductal adenocarcinoma tumor DNA. Transl. Cancer Res..

[42] Lee B., Lipton L., Cohen J., Tie J., Javed A.A., Li L., Goldstein D., Burge M., Cooray P., Nagrial A. (2019). Circulating tumor DNA as a potential marker of adjuvant chemotherapy benefit following surgery for localized pancreatic cancer. Ann. Oncol..

[43] Liu X., Liu L., Ji Y., Li C., Wei T., Yang X., Zhang Y., Cai X., Gao Y., Xu W. (2019). Enrichment of short mutant cell-free DNA fragments enhanced detection of pancreatic cancer. EBioMedicine.

[44] Guo S., Shi X., Shen J., Gao S., Wang H., Shen S., Pan Y., Li B., Xu X., Shao Z. (2020). Preoperative detection of KRAS G12D mutation in ctDNA is a powerful predictor for early recurrence of resectable PDAC patients. Br. J. Cancer.

[45] Watanabe F., Suzuki K., Tamaki S., Abe I., Endo Y., Takayama Y., Ishikawa H., Kakizawa N., Saito M., Futsuhara K. (2019). Longitudinal monitoring of KRAS-mutated circulating tumor DNA enables the prediction of prognosis and therapeutic responses in patients with pancreatic cancer. PLoS ONE.

[46] Lin M., Alnaggar M., Liang S., Chen J., Xu K., Dong S., Du D., Niu L. (2018). Circulating Tumor DNA as a Sensitive Marker in Patients Undergoing Irreversible Electroporation for Pancreatic Cancer. Cell Physiol. Biochem..

[47] Nakano Y., Kitago M., Matsuda S., Nakamura Y., Fujita Y., Imai S., Shinoda M., Yagi H., Abe Y., Hibi T. (2018). KRAS mutations in cell-free DNA from preoperative and postoperative sera as a pancreatic cancer marker: A retrospective study. Br. J. Cancer.

[48] Jiang J., Ye S., Xu Y., Chang L., Hu X., Ru G., Guo Y., Yi X., Yang L., Huang D. (2020). Circulating Tumor DNA as a Potential Marker to Detect Minimal Residual Disease and Predict Recurrence in Pancreatic Cancer. Front. Oncol..

[49] Sausen M., Phallen J., Adleff V., Jones S., Leary R.J., Barrett M.T., Anagnostou V., Parpart-Li S., Murphy D., Kay Li Q. (2015). Clinical implications of genomic alterations in the tumour and circulation of pancreatic cancer patients. Nat. Commun..

[50] Van Laethem J.L., Riess H., Jassem J., Haas M., Martens U.M., Weekes C., Peeters M., Ross P., Bridgewater J., Melichar B. (2017). Phase I/II Study of Refametinib (BAY 86-9766) in Combination with Gemcitabine in Advanced Pancreatic cancer. Target Oncol..

[51] Singh N., Rashid S., Dash N.R., Gupta S., Saraya A. (2020). Clinical significance of promoter methylation status of tumor suppressor genes in circulating DNA of pancreatic cancer patients. J. Cancer Res. Clin. Oncol..

[52] Bernard V., Kim D.U., San Lucas F.A., Castillo J., Allenson K., Mulu F.C., Stephens B.M., Huang J., Semaan A., Guerrero P.A. (2019). Circulating Nucleic Acids Are Associated With Outcomes of Patients With Pancreatic Cancer. Gastroenterology.

[53] Chen H., Tu H., Meng Z.Q., Chen Z., Wang P., Liu L.M. (2010). K-ras mutational status predicts poor prognosis in unresectable pancreatic cancer. Eur. J. Surg. Oncol..

[54] Mulcahy H.E., Lyautey J., Lederrey C., qi Chen X., Anker P., Alstead E.M., Ballinger A., Farthing M.J., Stroun M. (1998). A prospective study of K-ras mutations in the plasma of pancreatic cancer patients. Clin. Cancer Res..

[55] Earl J., Garcia-Nieto S., Martinez-Avila J.C., Montans J., Sanjuanbenito A., Rodríguez-Garrote M., Lisa E., Mendía E., Lobo E., Malats N. (2015). Circulating tumor cells (Ctc) and kras mutant circulating free Dna (cfdna) detection in peripheral blood as biomarkers in patients diagnosed with exocrine pancreatic cancer. BMC Cancer.

[56] Singh N., Gupta S., Pandey R.M., Chauhan S.S., Saraya A. (2015). High levels of cell-free circulating nucleic acids in pancreatic cancer are associated with vascular encasement, metastasis and poor survival. Cancer Investig..

[57] Adamo P., Cowley C.M., Neal C.P., Mistry V., Page K., Dennison A.R., Isherwood J., Hastings R., Luo J., Moore D.A. (2017). Profiling tumour heterogeneity through circulating tumour DNA in patients with pancreatic cancer. Oncotarget.

[58] Cheng H., Liu C., Jiang J., Luo G., Lu Y., Jin K., Guo M., Zhang Z., Xu J., Liu L. (2017). Analysis of ctDNA to predict prognosis and monitor treatment responses in metastatic pancreatic cancer patients. Int. J. Cancer.

[59] Pietrasz D., Pécuchet N., Garlan F., Didelot A., Dubreuil O., Doat S., Imbert-Bismut F., Karoui M., Vaillant J.C., Taly V. (2017). Plasma Circulating Tumor DNA in Pancreatic Cancer Patients Is a Prognostic Marker. Clin. Cancer Res..

[60] Kim M.K., Woo S.M., Park B., Yoon K.A., Kim Y.H., Joo J., Lee W.J., Han S.S., Park S.J., Kong S.Y. (2018). Prognostic Implications of Multiplex Detection of KRAS Mutations in Cell-Free DNA from Patients with Pancreatic Ductal Adenocarcinoma. Clin. Chem..

[61] Patel H., Okamura R., Fanta P., Patel C., Lanman R.B., Raymond V.M., Kato S., Kurzrock R. (2019). Clinical correlates of blood-derived circulating tumor DNA in pancreatic cancer. J. Hematol. Oncol..

[62] Bachet J.B., Blons H., Hammel P., Hariry I.E., Portales F., Mineur L., Metges J.P., Mulot C., Bourreau C., Cain J. (2020). Circulating Tumor DNA is Prognostic and Potentially Predictive of Eryaspase Efficacy in Second-line in Patients with Advanced Pancreatic Adenocarcinoma. Clin. Cancer Res..

[63] Strijker M., Soer E.C., de Pastena M., Creemers A., Balduzzi A., Beagan J.J., Busch O.R., van Delden O.M., Halfwerk H., van Hooft J.E. (2020). Circulating tumor DNA quantity is related to tumor volume and both predict survival in metastatic pancreatic ductal adenocarcinoma. Int. J. Cancer.

[64] Toledano-Fonseca M., Cano M.T., Inga E., Rodríguez-Alonso R., Gómez-España M.A., Guil-Luna S., Mena-Osuna R., de la Haba-Rodríguez J.R., Rodríguez-Ariza A., Aranda E. (2020). Circulating Cell-Free DNA-Based Liquid Biopsy Markers for the Non-Invasive Prognosis and Monitoring of Metastatic Pancreatic Cancer. Cancers.

[65] Uesato Y., Sasahira N., Ozaka M., Sasaki T., Takatsuki M., Zembutsu H. (2020). Evaluation of circulating tumor DNA as a biomarker in pancreatic cancer with liver metastasis. PLoS ONE.

[66] Wei T., Zhang J., Li J., Chen Q., Zhi X., Tao W., Ma J., Yang J., Lou Y., Ma T. (2020). Genome-wide profiling of circulating tumor DNA depicts landscape of copy number alterations in pancreatic cancer with liver metastasis. Mol. Oncol..

[67] Sugimori M., Sugimori K., Tsuchiya H., Suzuki Y., Tsuyuki S., Kaneta Y., Hirotani A., Sanga K., Tozuka Y., Komiyama S. (2020). Quantitative monitoring of circulating tumor DNA in patients with advanced pancreatic cancer undergoing chemotherapy. Cancer Sci..

[68] Snyder M., Kircher M., Hill A., Daza R., Shendure J. (2016). Cell-free DNA Comprises an In Vivo Nucleosome Footprint that Informs Its Tissues-Of-Origin. Cell.

[69] Fernando M.R., Jiang C., Krzyzanowski G.D., Ryan W.L. (2018). Analysis of human blood plasma cell-free DNA fragment size distribution using EvaGreen chemistry based droplet digital PCR assays. Clin. Chim. Acta.

[70] Li H., Jing C., Wu J., Ni J., Sha H., Xu X., Du Y., Lou R., Dong S., Feng J. (2019). Circulating tumor DNA detection: A potential tool for colorectal cancer management. Oncol. Lett..

[71] Lee J.S., Rhee T.M., Pietrasz D., Bachet J.B., Laurent-Puig P., Kong S.Y., Takai E., Yachida S., Shibata T., Lee J.W. (2019). Circulating tumor DNA as a prognostic indicator in resectable pancreatic ductal adenocarcinoma: A systematic review and meta-analysis. Sci. Rep..

[72] Vesovic N., Tosic N., Djurasevic T.K., Andric Z., Zdravkovic D., Pavlovic S., Jovanovic D. (2020). Expression pattern of circulating long non-coding RNA GAS5 as a novel biomarker in non-small cell lung cancer patients. Arch. Med. Sci..

[73] O’Leary B., Hrebien S., Beaney M., Fribbens C., Garcia-Murillas I., Jiang J., Li Y., Huang Bartlett C., André F., Loibl S. (2019). Comparison of BEAMing and Droplet Digital PCR for Circulating Tumor DNA Analysis. Clin. Chem..

[74] Pécuchet N., Rozenholc Y., Zonta E., Pietrasz D., Didelot A., Combe P., Gibault L., Bachet J.B., Taly V., Fabre E. (2016). Analysis of Base-Position Error Rate of Next-Generation Sequencing to Detect Tumor Mutations in Circulating DNA. Clin. Chem..

[75] Heitzer E., Ulz P., Geigl J.B. (2015). Circulating tumor DNA as a liquid biopsy for cancer. Clin Chem..

[76] Trigg R.M., Martinson L.J., Parpart-Li S., Shaw J.A. (2018). Factors that influence quality and yield of circulating-free DNA: A systematic review of the methodology literature. Heliyon.

[77] Mullard A. (2019). Cracking KRAS. Nat. Rev. Drug Discov..

[78] Waters A.M., Der C.J. (2018). KRAS: The Critical Driver and Therapeutic Target for Pancreatic Cancer. Cold Spring Harb. Perspect Med..

[79] Haigis K.M. (2017). KRAS Alleles: The Devil Is in the Detail. Trends Cancer.

[80] Gillson J., Ramaswamy Y., Singh G., Gorfe A.A., Pavlakis N., Samra J., Mittal A., Sahni S. (2020). Small Molecule KRAS Inhibitors: The Future for Targeted Pancreatic Cancer Therapy?. Cancers.

[81] Pishvaian M.J., Blais E.M., Brody J.R., Lyons E., DeArbeloa P., Hendifar A., Mikhail S., Chung V., Sahai V., Sohal D.P.S. (2020). Overall survival in patients with pancreatic cancer receiving matched therapies following molecular profiling: A retrospective analysis of the Know Your Tumor registry trial. Lancet Oncol..

[82] Takai E., Totoki Y., Nakamura H., Morizane C., Nara S., Hama N., Suzuki M., Furukawa E., Kato M., Hayashi H. (2015). Clinical utility of circulating tumor DNA for molecular assessment in pancreatic cancer. Sci. Rep..

[83] Botrus G., Kosirorek H., Sonbol M.B., Kusne Y., Uson Junior P.L.S., Borad M.J., Ahn D.H., Kasi P.M., Drusbosky L.M., Dada H. (2021). Circulating Tumor DNA-Based Testing and Actionable Findings in Patients with Advanced and Metastatic Pancreatic Adenocarcinoma. Oncologist.

[84] Heredia-Soto V., Rodríguez-Salas N., Feliu J. (2021). Liquid Biopsy in Pancreatic Cancer: Are We Ready to Apply It in the Clinical Practice?. Cancers.

[85] Chan H.T., Chin Y.M., Nakamura Y., Low S.K. (2020). Clonal Hematopoiesis in Liquid Biopsy: From Biological Noise to Valuable Clinical Implications. Cancers.

[86] Jaiswal S., Fontanillas P., Flannick J., Manning A., Grauman P.V., Mar B.G., Lindsley R.C., Mermel C.H., Burtt N., Chavez A. (2014). Age-related clonal hematopoiesis associated with adverse outcomes. N. Engl. J. Med..

[87] Ptashkin R.N., Mandelker D.L., Coombs C.C., Bolton K., Yelskaya Z., Hyman D.M., Solit D.B., Baselga J., Arcila M.E., Ladanyi M. (2018). Prevalence of Clonal Hematopoiesis Mutations in Tumor-Only Clinical Genomic Profiling of Solid Tumors. JAMA Oncol..

[88] Manrai A.K., Funke B.H., Rehm H.L., Olesen M.S., Maron B.A., Szolovits P., Margulies D.M., Loscalzo J., Kohane I.S. (2016). Genetic Misdiagnoses and the Potential for Health Disparities. N. Engl. J. Med..

[89] Lek M., Karczewski K.J., Minikel E.V., Samocha K.E., Banks E., Fennell T., O’Donnell-Luria A.H., Ware J.S., Hill A.J., Cummings B.B. (2016). Analysis of protein-coding genetic variation in 60,706 humans. Nature.

[90] Karczewski K.J., Francioli L.C., Tiao G., Cummings B.B., Alföldi J., Wang Q., Collins R.L., Laricchia K.M., Ganna A., Birnbaum D.P. (2020). The mutational constraint spectrum quantified from variation in 141,456 humans. Nature.

