# Supplementary material for: Meta-Analysis of Circulating Cell-Free DNA’s Role in the Prognosis of Pancreatic Cancer
Source: Cancers (Basel). 2021 Jul 6;13(14):3378. doi: 10.3390/cancers13143378 (PMC8303288; doi:10.3390/cancers13143378)
Supplement: Supplementary file 1 [file cancers-13-03378-s001.zip › cancers-1246547-SI.pdf]

cancers

Systematic Review

Meta-Analysis of Circulating Cell-Free DNA's Role in the Prognosis of Pancreatic Cancer

Jelena Milin-Lazovic [1,†], Petar Madzarevic [1,†], Nina Rajovic [1], Vladimir Djordjevic [2,3], Nikola Milic [3], Sonja Pavlovic [4], Nevena Veljkovic [5,6], Natasa M. Milic [1,7,‡] and Dejan Radenkovic [2,3,*,‡]

1 Institute for Medical Statistics and Informatics, Faculty of Medicine, University of Belgrade, 11000 Belgrade, Serbia; jelena.milin@med.bg.ac.rs (J.M.-L.); petar.madzarevic@gmail.com (P.M.); nina.rajovic@med.bg.ac.rs (N.R.); milic.natasa@mayo.edu (N.M.M.)
2 Department of Surgery, University Clinical Center of Serbia, 11000 Belgrade, Serbia; vladimir.djordjevic@kcs.ac.rs
3 Faculty of Medicine, University of Belgrade, 11000 Belgrade, Serbia; nmilic1996@gmail.com
4 Institute of Molecular Genetics and Genetic Engineering, University of Belgrade, 11000 Belgrade, Serbia; sonya@imgge.bg.ac.rs
5 Vinca Institute of Nuclear Sciences, National Institute of the Republic of Serbia, University of Belgrade, 11000 Belgrade, Serbia; nevena.veljkovic@heliant.rs
6 Heliant Ltd, 11000 Belgrade, Serbia
7 Department of Internal Medicine, Division of Nephrology and Hypertension, Mayo Clinic, Rochester, MN 55902, USA
* Correspondence: dejan.radenkovic@med.bg.ac.rs
† First co-authors.
‡ Senior co-authors.

Citation: Milin-Lazovic, J; Madzarevic, P; Rajovic, N; Djordjevic, P; Milic, N; Pavlovic, S; Veljkovic, N; Milic, M.N.; Radenkovic, D. Meta-Analysis of Circulating Cell-Free DNA's Role in the Prognosis of Pancreatic Cancer. *Cancers* 2021, *13*, 3378. https://doi.org/10.3390/cancers13143378

Academic Editors: Sohei Satoi and Inna Chervoneva

Received: 19 May 2021
Accepted: 23 June 2021
Published: 6 July 2021

Publisher's Note: MDPI stays neutral with regard to jurisdictional claims in published maps and institutional affiliations.

Copyright: © 2021 by the authors. Submitted for possible open access publication under the terms and conditions of the Creative Commons Attribution (CC BY) license (http://creativecommons.org/licenses/by/4.0/).

Supplementary

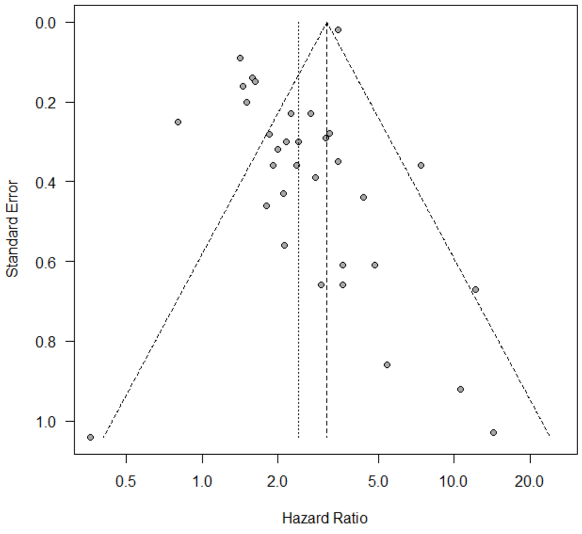

Figure S1. Funnel plot of the meta-analysis presented in Figure 3.

Study or Subgroup	log[Hazard Ratio]	SE	experimental Total	control Total	Weight	Hazard Ratio IV, Random, 95% CI
Ako G12D, plasma, 2017	0.59	0.46	15	25	2.7%	1.80 [0.73, 4.44]
Ako G12V, plasma, 2017	2.66	1.03	5	35	1.0%	14.30 [1.90, 107.64]
Bachet, 2020	0.65	0.36	77	36	3.3%	1.92 [0.95, 3.88]
Bernard, 2020	0.86	0.36	70	34	3.3%	2.36 [1.17, 4.79]
Castells, 1999	0.41	0.2	12	32	4.4%	1.51 [1.02, 2.23]
Chen, 2010	2	0.36	30	61	3.3%	7.39 [3.65, 14.96]
Chen, 2017	0.35	0.09	90	81	4.9%	1.42 [1.19, 1.69]
Cheng, 2017	0.37	0.16	65	123	4.6%	1.45 [1.06, 1.98]
Cheng, G12D, 2020	0.46	0.14	93	117	4.7%	1.58 [1.20, 2.08]
Cheng, G12V, 2020	0.48	0.15	61	149	4.7%	1.62 [1.20, 2.17]
Earl, 2015	2.5	0.67	8	23	1.8%	12.18 [3.28, 45.29]
Groot, 2019	1.03	0.39	29	30	3.2%	2.80 [1.30, 6.02]
Hadano, 2016	1.16	0.28	33	72	3.9%	3.19 [1.84, 5.52]
Lapin, 2018	0.88	0.3	31	30	3.7%	2.41 [1.34, 4.34]
Lee, 2019	1.28	0.66	23	14	1.8%	3.60 [0.99, 13.11]
Lin, 2018	1.13	0.29	20	45	3.8%	3.10 [1.75, 5.47]
Liu, 2019	0.74	0.43	51	23	2.9%	2.10 [0.90, 4.87]
Mohan, 2019	1.24	0.02	14	41	5.1%	3.46 [3.32, 3.59]
Mulcahy, 1998	1.28	0.61	17	4	2.0%	3.60 [1.09, 11.89]
Patel, 2019	1.47	0.44	50	44	2.9%	4.35 [1.84, 10.30]
Perets, 2018	1.69	0.86	5	12	1.3%	5.42 [1.00, 29.24]
Pietrasz, 2017	0.81	0.23	50	54	4.2%	2.25 [1.43, 3.53]
Sefrioui, 2017	0.69	0.32	28	15	3.6%	1.99 [1.06, 3.73]
Semrad, 2015	1.58	0.61	10	17	2.0%	4.85 [1.47, 16.05]
Singh, 2015	-0.22	0.25	34	76	4.1%	0.80 [0.49, 1.31]
Strijker, 2020	0.77	0.3	26	32	3.7%	2.16 [1.20, 3.89]
Tjensvol, 2016	1.09	0.66	10	4	1.8%	2.97 [0.82, 10.84]
Toledano, 2020	1.24	0.35	47	14	3.4%	3.46 [1.74, 6.86]
Ueasato, 2020	0.99	0.23	52	52	4.2%	2.69 [1.71, 4.22]
Wanatabe, 2018	0.75	0.56	21	57	2.2%	2.12 [0.71, 6.34]
Yang, 2018	2.36	0.92	10	25	1.1%	10.59 [1.75, 64.27]
**Total (95% CI)**			**1087**	**1377**	**100.0%**	**2.49 [2.00, 3.10]**

Heterogeneity: Tau² = 0.25; Chi² = 238.57, df = 30 (P < 0.00001); I² = 87%
Test for overall effect: Z = 8.13 (P < 0.00001)

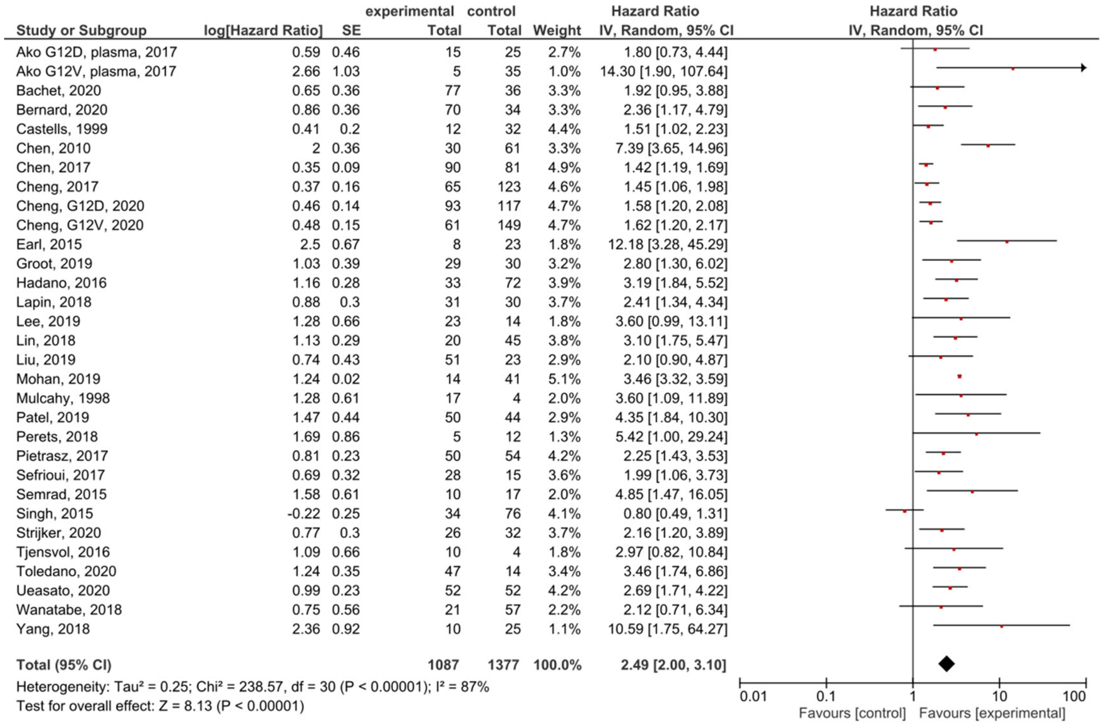

Figure S2. Forest plot presenting the relationship between presence of KRAS mutations before treatment in PDAC patients and OS: sensitivity analysis excluding samples from serum.

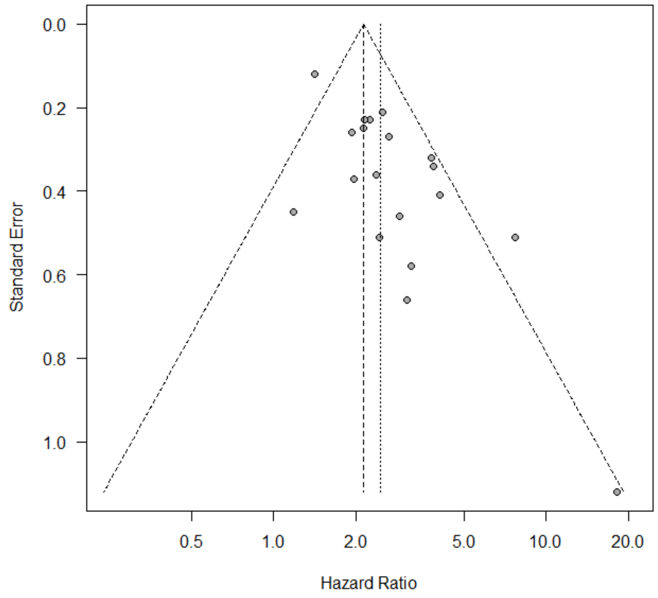

Figure S3. Funnel plot of the meta-analysis presented in Figure 4.

Study or Subgroup	log[Hazard Ratio]	SE	Experimental Total	Control Total	Weight	Hazard Ratio IV, Random, 95% CI
Bachet, 2020	0.76	0.25	77	36	9.3%	2.14 [1.31, 3.49]
Bernard, 2020	0.66	0.26	70	34	9.0%	1.93 [1.16, 3.22]
Chen, 2017	0.35	0.12	54	57	14.7%	1.42 [1.12, 1.80]
Kim, 2018	0.97	0.27	38	23	8.6%	2.64 [1.55, 4.48]
Lapin, 2018	1.33	0.32	31	30	7.1%	3.78 [2.02, 7.08]
Nakano, 2018	0.17	0.45	20	25	4.5%	1.19 [0.49, 2.86]
Pietrasz, 2017	0.81	0.23	50	54	10.1%	2.25 [1.43, 3.53]
Sausen, 2015	0.87	0.36	22	28	6.2%	2.39 [1.18, 4.83]
Semrad, 2015	0.89	0.51	10	17	3.7%	2.44 [0.90, 6.62]
Sigumori, 2020	0.68	0.37	19	87	5.9%	1.97 [0.96, 4.08]
Tjensvol, 2016	1.13	0.66	10	4	2.4%	3.10 [0.85, 11.29]
Toledano, 2020	1.35	0.34	42	14	6.6%	3.86 [1.98, 7.51]
Ueasato, 2020	0.92	0.21	48	56	10.8%	2.51 [1.66, 3.79]
Yang, 2018	2.9	1.12	10	25	0.9%	18.17 [2.02, 163.23]
**Total (95% CI)**			**501**	**490**	**100.0%**	**2.27 [1.83, 2.82]**

Heterogeneity: Tau² = 0.07; Chi² = 24.48, df = 13 (P = 0.03); I² = 47%
Test for overall effect: Z = 7.42 (P < 0.00001)

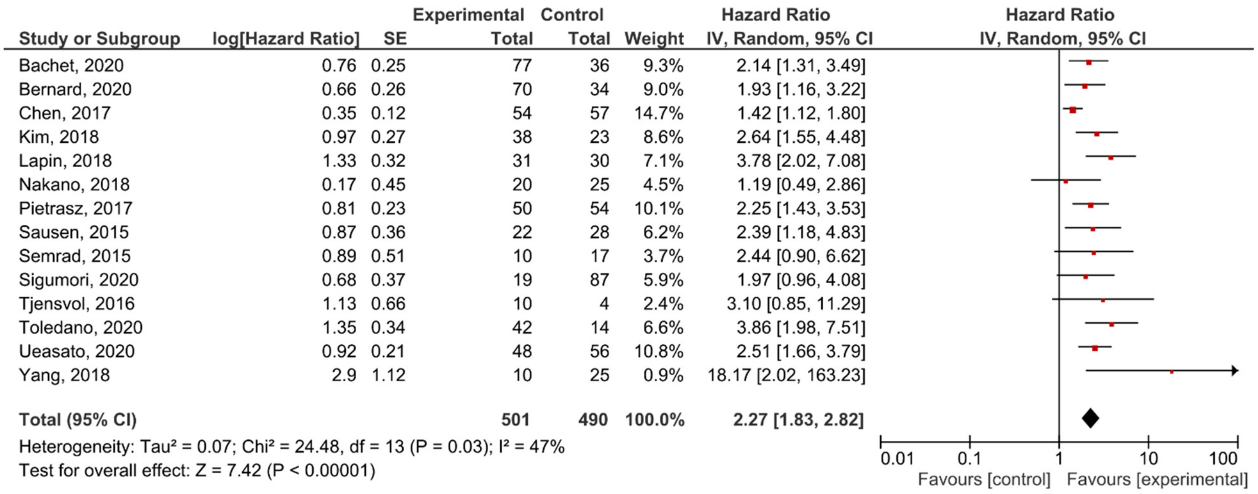

Figure S4. Forest plot presenting the relationship between presence of KRAS mutations before treatment in PDAC patients and PFS: sensitivity analysis including only PFS but not DFS and DSS.

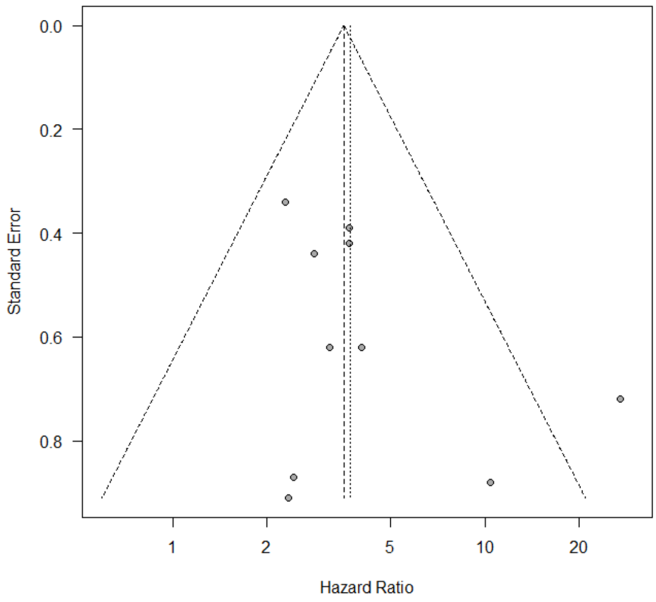

Figure S5. Funnel plot of the meta-analysis presented in Figure 5.

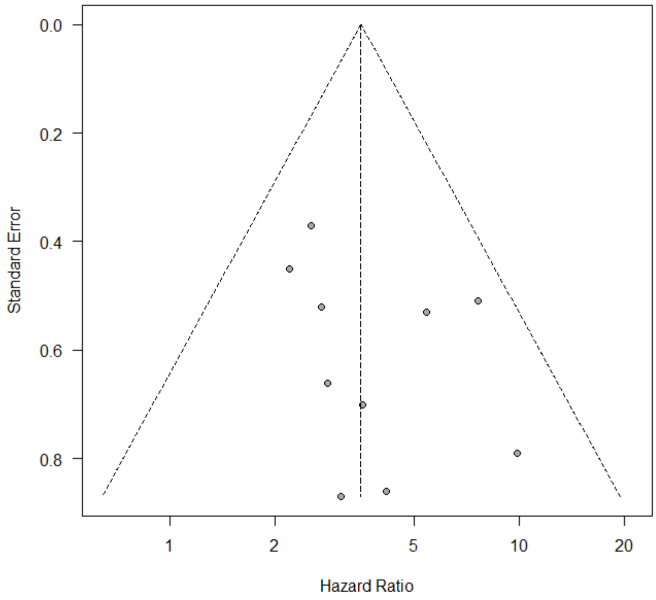

Figure S6. Funnel plot of the meta-analysis presented in Figure 6.

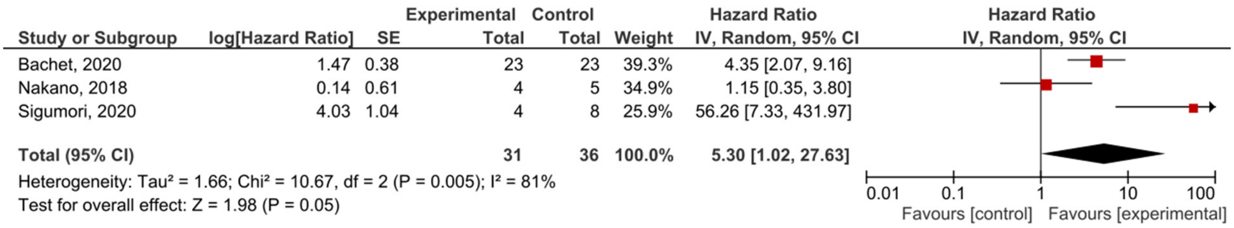

Figure S7. Forest plot comparing PFS between responders (patients who were KRAS positive before treatment and KRAS negative after treatment) and non-responders (patients who were KRAS positive before treatment and remained KRAS positive after the treatment).

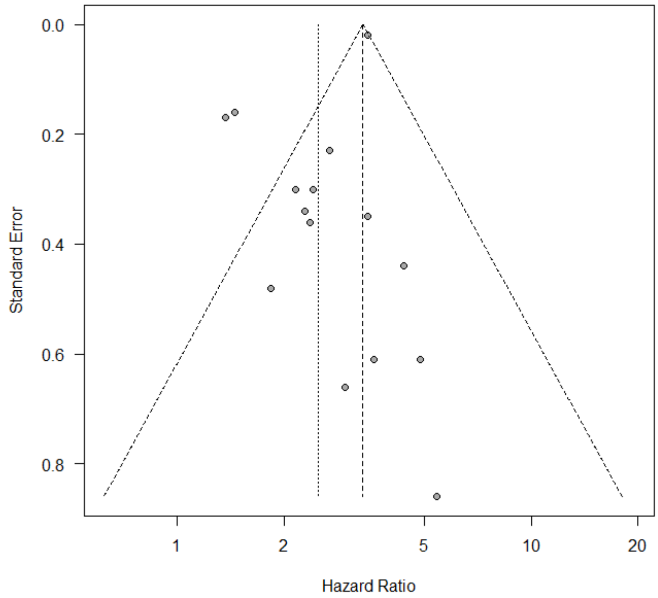

Figure S8. Funnel plot of the meta-analysis presented in Figure 7.

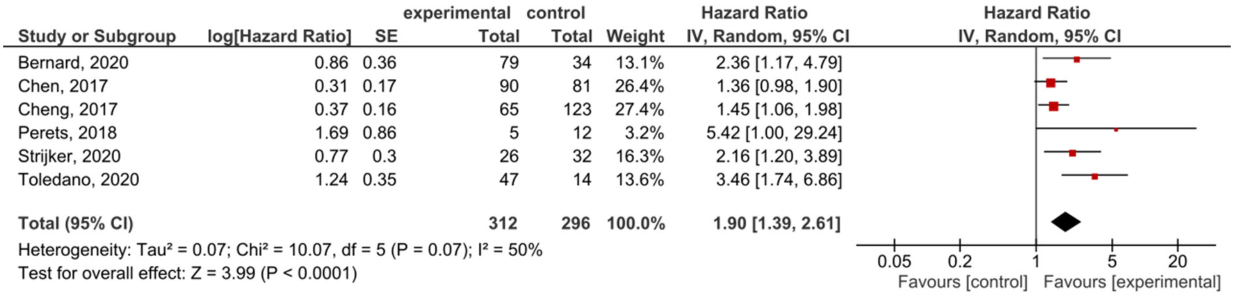

Figure S9. Forest plot presenting the relationship between presence of KRAS mutations before treatment in metastatic PDAC patients and OS.

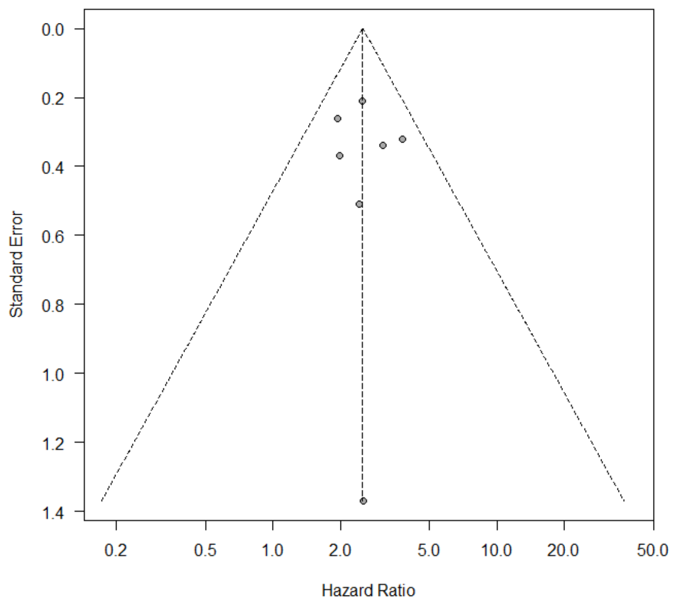

Figure S10. Funnel plot of the meta-analysis presented in Figure 8.
